# gga-miR-200b-3p Promotes Macrophage Activation and Differentiation *via* Targeting Monocyte to Macrophage Differentiation-Associated in HD11 Cells

**DOI:** 10.3389/fimmu.2020.563143

**Published:** 2020-09-30

**Authors:** Wencheng Lin, Lianghui Zhou, Manqing Liu, Danmeng Zhang, Yiming Yan, Yung-Fu Chang, Xiquan Zhang, Qingmei Xie, Qingbin Luo

**Affiliations:** ^1^ College of Animal Science, South China Agricultural University, Guangzhou, China; ^2^ Guangdong Engineering Research Center for Vector Vaccine of Animal Virus, Guangzhou, China; ^3^ Guangdong Provincial Key Lab of Agro-Animal Genomics and Molecular Breeding & Key Laboratory of Chicken Genetics, Breeding and Reproduction, Ministry of Agriculture, Guangzhou, China; ^4^ Department of Population Medicine and Diagnostic Sciences, College of Veterinary Medicine, Cornell University, Ithaca, NY, United States

**Keywords:** gga-miR-200b-3p, macrophage, activation, MMD, cytokine

## Abstract

MicroRNAs (miRNAs) play a critical role in various biological processes through regulation of gene expression post-transcriptionally. Although miRNAs are involved in cell proliferation and differentiation in mammals, few reports regarding the effects of host miRNAs on macrophage activation and differentiation are available in birds. Here, we reported that gga-miR-200b-3p acts as a positive regulator, enhancing macrophage activation and differentiation using an avian model. We found that ectopic expression of gga-miR-200b-3p in HD11 cells enhances the amount of MHC-II-positive cells and promotes the expression of pro-inflammatory cytokines and that gga-miR-200b-3p directly targets monocyte to macrophage differentiation-associated (MMD). The inhibition of MMD by gga-miR-200b-3p enhances the activation and differentiation of HD11 cells and increases the expression of pro-inflammatory cytokines. Collectively, these findings highlight a crucial role of gga-miR-200b-3p in macrophage activation and differentiation in birds.

## Introduction

MicroRNAs (miRNAs) are a family of evolutionarily conserved noncoding RNAs, ranging from 18 to 24 nt in length. MiRNAs can be found as isolated transcript units or clustered and co-transcribed as polycistronic primary transcripts (1), regulating the expression of target genes post-transcriptionally through degradation of mRNAs or inhibition of translation by binding to complementary sites in the 3′UTRs of mRNAs (2–4). MiRNAs usually serve as guide RNAs to target mRNAs bearing complementary sequences, which is essential for the specificity of miRNA-target mRNA interaction (5). Yet, the specificity of miRNA-target mRNA interaction is also involved in the presence and cooperation between multiple target sites (TSs), the spacing between TSs, position within the mRNA, proximity to the stop codon, and the target mRNA secondary structures (6, 7). All multicellular organisms and certain viruses produce miRNAs. MiRNAs are playing a wide variety of roles in cancer development and differentiation (8), cell proliferation and differentiation (9), cell cycle and apoptosis (10), immunoregulation, and viral infection (11, 12).

The miR-200 family comprises miR-141, miR-200a, miR-200b, miR-200c, and miR-429 (13), which are highly homologous and derived from two gene clusters. miR-200a, miR-200b, and miR-429 originated from chromosome 1p33.36; miR-200c and miR-141 are derived from chromosome 12p13.3 (14). Previous studies indicated that the miR-200 family involved the regulation of epithelial-mesenchymal transition (EMT) and mesenchymal-to-epithelial transition, neoplastic transformation, cancer cell proliferation, and drug resistance (15–18), providing evidence that the miR-200 family is a family of tumor suppressor miRNAs.

MiR-200b-3p has been confirmed to play critical roles in many biological processes through regulating target genes. The previous study indicated that miR-200b-3p promoted G1/S arrest and monocyte/macrophage differentiation through enhancing the activity of p38 MAPK in humans (19). However, few reports regarding the effects of host miRNAs on the activation and differentiation of macrophage in birds are available, the impact of miR-200b-3p on macrophage in birds is still unclear. In this study, we screened the spleens of vaccine-immunized chickens for the potential host miRNAs regulating the activation and differentiation of macrophages. We found that gga-miR-200b-3p effectively enhances macrophage activation and differentiation *via* targeting the monocyte to macrophage differentiation-associated (MMD). Thus, gga-miR-200b-3p plays a critical role in macrophage activation and differentiation in birds.

## Materials and Methods

### Ethics Statement

This study was approved by the Animal Care Committee of South China Agricultural University (approval ID: SYXK-2014-0136). All study procedures and animal care activities were conducted per the recommendations in the Guide for the Care and Use of Laboratory Animals of the Ministry of Science and Technology of the People’s Republic of China.

### Cells and Animals

HD11 cells (20) were kindly provided by Dr. Liu Jue at the Beijing Academy of Agriculture and Forestry and cultured in RPMI 1640 supplemented with 10% fetal bovine serum (FBS). DF-1 cells were cultured in Dulbecco modified Eagle medium (DMEM) containing 10% FBS. The specific‐pathogen‐free (SPF) chickens were purchased from the Guangdong DHN Poultry and Egg Products Co. Ltd., China.

### Small RNA Sequencing Analysis

A total of six -day-old SPF chickens were assigned to two groups (three chickens per group). Chickens in the experimental group were immunized with an inactivated NDV vaccine (Yebio, China), while chickens in the other group were immunized with PBS. All the chickens were euthanized to collect spleens at 5 days post-immunization. High-quality total RNAs were isolated from spleens using TRIzol reagent (Invitrogen), and miRNA sequencing was performed by LC Sciences (Hangzhou).

### miRNA Mimics and miRNA Target Prediction

MiRNA mimics and inhibitors were synthesized by GenePharma Company (Shanghai). The sense sequences were as follows: for gga-miR-200b-3p mimics, 5′-UAAUACUGCCUGGUAAUGAUGAU-3′; for gga-miR-200b-3p inhibitors, 5′-AUUAUGACGGACCAUUACUACUA-3′; for the negative control mimics, 5′-UUUGUACUACACAAAAGUACUG -3′; and for negative-control inhibitors, 5′- UUUGUACUACACAAAAGUACUG-3′. MiRNA targets in the host were predicted by the use of Diana Tools (http://diana.imis.athena-innovation.gr/DianaTools/index.php?r=microT_CDS/index), PicTar (http://www.pictar.org/), Targetscan (http://www.targetscan.org/vert_72/), and miRDB (http://mirdb.org/).

### Construction of Plasmids

Specific primer pairs for the gga-miR-200b-3p target sequence in MMD were designed as follows: for the gga-miR-200b-3p target sequence at position 37-43 in MMD, sense primer 5′-CCCTCGAGCTTGGCGTGGTGTTCTTC-3′ and antisense primer 5′-TGCTCTAGATACAGCACTTGCCGTCTG-3′; for the gga-miR-200b-3p target sequence at position 934-940 in MMD, sense primer 5′-CCCTCGAG CTTTACTTGTATTGCTGTC-3′ and antisense primer 5′-TGCTCTAGA GTTGGAAGATAGATAGGC-3′. The seed sites mutant in MMD gene were constructed by mutating nucleotides in the seed sequences (Position 37-43 in MMD: 5′-GCTGCACTTGGCCTCTGTAA***CAGTATT***ATTTGACTTAAGGAA-3′ mutated to 5′-GCTGCACTTGGCCTCTGTAA***GCTCGCC***ATTTGACTTAAGGAA-3′; Position 934-940 in MMD: 5′-ATTTACTCAGTTTTTCATGT***CAGTATT***TGAAAACAAAACCAA-3′ mutated to 5′-ATTTACTCAGTTTTTCATGT***TGTCCGG***TGAAAACAAAACCAA-3′) using a fast mutagenesis system (Transgene, China). All the primers were synthesized by Sangon Company (China).

All the fragments were amplified using a high-fidelity pfu DNA polymerase (Q5™ High-Fidelity DNA polymerase, NEB) under the following conditions: 98°C for 10 s, 40 cycles of 98°C for 10 s, 56°C for 30 s, and 72°C for 30 s, and 72°C for 10 min. The PCR products were cloned into a pEASY-Blunt plasmid (TransGen Biotech, Beijing) and sequenced commercially (Songon Biotech, Shanghai). The fragments of the MMD gene around the predicted gga-miR-200b-3p target sites were inserted into the site downstream of the firefly luciferase gene in the pGL3-Control vector to create the wild type or mutant 3′UTR vector.

### Quantitative Real-Time PCR (qRT-PCR)

HD11 cells were transfected with miRNA mimics, inhibitors, or controls using Lipofectamine 3000 reagent (Invitrogen). Twenty-four hours after transfection, cells were harvested for quantification of gene expression. Briefly, the specific primer pairs for chicken CD11b, F4/80, MHC-II, TNF-α, IL-1β, IL-6, IL-12α, CD14, CD68, RANTES, MIP-1, MMD, and GAPDH were designed according to the previous reports (**Table 1**). Total RNA was isolated using TRIzol reagent and subjected to qRT-PCR detection. GAPDH was utilized as the reference gene. The transcriptional levels of genes were calculated relative to that of the GAPDH and are presented as fold increases or decreases relative to the control sample level.

**Table 1:** Primer pairs used in this study.

Gene	Primer pairs (5’ to 3’)	Reference
*CD11b*	F: CAAATCCCGCTCCGAAAGGC	(21)
	R: GCTCCCAAACAACCACCCCAC	
*F4/80*	F: GCACCATCTTGCTGGAGACT	Designed
	R: CTGGGGCCCCTGTAGATACT	
*MHC-II*	F: CTCGAGGTCATGATCAGCAA	(22)
	R: TGTAAACGTCTCCCCTTTGG	
*IL1β*	F: CAAGGTGACGGAGGAGGAC	(23)
	R: TAAATACCTCCACCCCGACA	
*IL6*	F: GCTTCGACGAGGAGAAATGC	(24)
	R: GCCAGGTGCTTTGTGCTGTA	
*IL12α*	F: AGACGTCACCAACAGTCAGAG	Designed
	R: CAGATCCTTGAGGTTCCCCAG	
*CD14*	F: GGACGACTCCACCATTGACAT	(25)
	R: GGAGGACCTCAGGAACCAGAA	
*CD68*	F: AGCCTTGTGTTCAGCTCCAA	Designed
	R: TCCCCTGGACCTTGGTTTTG	
*MIP-1*	F: CCTGCTGCTTGTCCTACG	Designed
	R: GGCGGCATTTGCTGCTGG	
*RANTES*	F: CTCCGTTTGGGGCTGATACA	Designed
	R: GATGAACACAACTGCTGCCTG	
*TNF-α*	F: CGCTCAGAACGACGTCAA	(24)
	R: GTCGTCCACACCAACGAG	
*MMD*	F: GCTAACAGCCGCTACAAA	Designed
	R: GAAAGTCGGTGGAGAAGG	
*GAPDH*	F: TGCCATCACAGCCACACAGAAG	(26)
	R: ACTTTCCCCACAGCCTTAGCAG	

### Dual-Luciferase Reporter Gene Assay

DF-1 cells were transfected with luciferase reporter plasmids (pGL3-MMD-wt or pGL3-MMD-mut) and miRNA mimics, inhibitors or controls. pRL-TK Renilla luciferase reporter plasmid was used as the reference control to normalize the transfection efficiency. Forty-eight hours after transfection, relative luciferase activities were detected using a dual-luciferase assay kit (Promega, USA).

### Knockdown of MMD by RNA Interference (RNAi)

siRNAs were designed by GenePharma Co. (Shanghai, China) to knock down MMD in this study. The siRNAs for targeting MMD in HD11 cells included the followings: RNAi#1 (sense, 5′-ACACGCATTCCTCATTGtt-3′; antisense, 5′- CTTUGAGGAAUGCGUGUtt -3′), RNAi#2 (sense, 5′-AGACATCATTCGTCACTTAtt-3′; antisense, 5′-UAAGUGACGAAUGAUGUCUtt-3′), RNAi#3 (sense, 5′-CCACAGTGTTTCATATCGTtt-3′; antisense, 5′-ACGAUAUGAAACACUGUGGtt-3′), and negative control (sense, 5′-UUCUCCGAACGUGUCACGUtt-3′; antisense, 5′-ACGUGACACGUUCGGAGAAtt-3′). HD11 cells were transfected with siRNA or controls twice at a 20 h interval. Twenty-four hours after the second transfection, cells were harvested for further analysis.

### Preparation of Polyclonal Antibodies

Polyclonal antibodies against chMMD were prepared in mice and used to investigate the expression of MMD in HD11 cells. Briefly, 8-weeks-old BALB/c mice were immunized with chMMD protein for three times at 3-weeks intervals. The blood samples were collected from the orbital sinus and processed to collect sera for the detection of anti-chMMD antibodies by indirect ELISA and Western Blot.

### Western Blot Analysis

Western blot was used to examine the effect of gga-miR-200b-3p and knockdown of MMD on the expression of MHC-II in HD11 cells. The HD11 cells were firstly transfected with miRNA mimics, control miRNA, MMD-specific siRNA or control siRNA, respectively. LPS-treated HD11 cells were used as a positive control. Twenty-four hours after transfection, cells were lysed and centrifuged for sample collection. Samples were fractionated by SDS–PAGE and subsequently transferred to a polyvinylidene difluoride membrane, the followingblock with 5% skimmed milk and probe with indicated antibodies. Blots were developed using an ECL kit (ThermoFisher, USA).

### Flow Cytometry

HD11 cells were transfected with miRNA mimics or control miRNA, or MMD-specific siRNA or control siRNA. LPS-treated HD11 cells were used as a positive control. Cells were harvested and stained with FITC-conjugated anti-Chicken MHC-II (8350-09, SouthernBiotech). The cell fluorescence was examined with flow cytometry and analyzed with CellQuest software (BD Biosciences).

### ELISA

HD11 cells were transfected with miRNA mimics or control miRNA, or MMD-specific siRNA or control siRNA. LPS-treated HD11 cells were used as a positive control. The cell culture supernatants were collected at 24 h after transfection or treatment. The concentrations of TNF-α, IL-1β, IL-6 and IL-12α in cell culture supernatants were quantified using sandwich ELISA kits (Cloud-Clone).

### Detection of Intracellular Reactive Oxygen Species (ROS)

Intracellular ROS was detected by measuring the oxidative conversion of permeable 2′, 7′-dichlorofluorescein diacetate (DCFH-DA) to fluorescent dichlorofluorescein (DCF) using a commercial kit (Beyotime, China). Briefly, HD11 cells were transfected with miRNA mimics or control miRNA, or MMD-specific siRNA or control siRNA. LPS-treated HD11 cells were used as a positive control. After incubation with DCFH-DA for 20 min, cells were harvested to detect DCF fluorescence distribution an excitation wavelength of 488 nm and at an emission wavelength of 525 nm.

### Statistical Analysis

The significance of differences between miRNA mimics-treated cells and controls in gene expression, cell activation, or intracellular ROS levels, between miRNA mimics-treated cells and mutant controls in gene expression, between MMD RNAi cells and controls in gene expression, cell activation, or intracellular ROS levels was determined by Mann-Whitney test or analysis of variance, respectively.

## Results

### miRNA Expression Profiles

**Figure 1:**
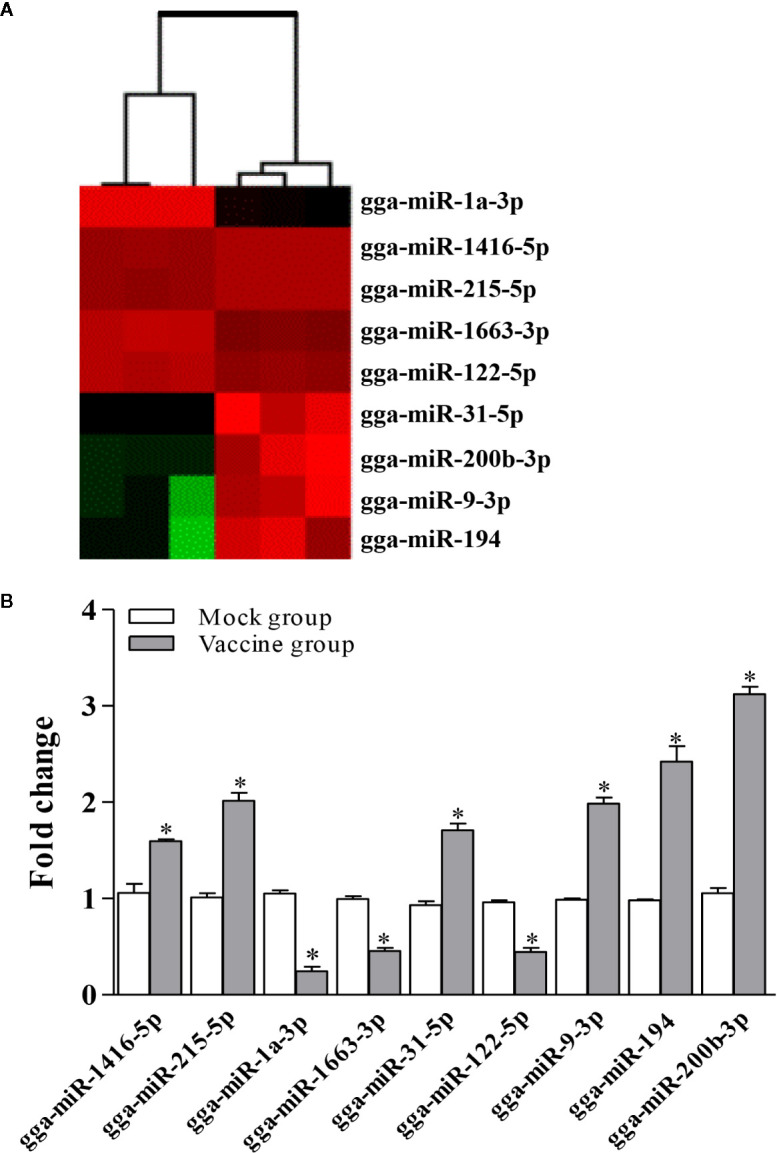
Screening for miRNAs affecting host immunity response. **(A)** Heat map of the candidate miRNAs. The heat map illustrates the expression profiles of 9 miRNAs in the spleen of vaccine- and mock-immunized chickens. **(B)** Validation of differentially expressed miRNAs using qRT-PCR. Expression levels of miRNAs were calculated relative to the expression of the GAPDH gene and expressed as fold increase or decrease relative to the control samples. Results are representative of three independent experiments and presented as mean ± SD. * stands for *P* < 0.05.

To identify the potential host miRNAs regarding the activation and differentiation of macrophage in birds, we screened the spleens of vaccine-immunized chickens and mock-immunized chickens by deep sequencing. As a result, 514 and 494 miRNAs were identified from the vaccine-immunized and mock-immunized chickens, respectively. Compared to that of mock-immunized chickens, 43 differentially expressed miRNAs were screened in vaccine-immunized chickens (threshold: *P* < 0.05 and fold change ≥ 2), including 25 up-regulated miRNAs and 18 down-regulated miRNAs. Among these differentially expressed miRNAs, a total of 9 miRNAs were selected for further study according to the functional analysis and the threshold value (**Figure 1A**), including three down-regulated miRNAs (gga-miR-1a-3p, gga-miR-122-5p and gga-miR-1663-3p) and six up-regulated miRNAs (gga-miR-1416-5p, gga-miR-122-5p, gga-miR-9-3p, gga-miR-200b-3p, gga-miR-31-5p and gga-miR-194).

Although miRNA-Seq possesses the ability to measure transcriptional levels, the accuracy of miRNA-Seq analysis needed to be verified using different methods. Therefore, we determined the differential expression of miRNAs mentioned above using qRT-PCR assay. As a result, compared to that in the mock-immunized chickens, the transcriptional levels of gga-miR-1a-3p, gga-miR-122-5p and gga-miR-1663-3p were significantly decreased in the vaccine-immunized chickens. In contrast, the transcriptional levels of gga-miR-1416-5p, gga-miR-122-5p, gga-miR-9-3p, gga-miR-200b-3p, gga-miR-31-5p, and gga-miR-194 were upregulated considerably (**Figure 1B**), yielding change trends similar to that determined by miRNA-Seq. These data provided solid evidence for the validity of miRNA-Seq analysis in the whole variations of the expression of miRNAs.

### gga-miR-200b-3p Enhances the Macrophage Activation and Differentiation in Birds

**Figure 2:**
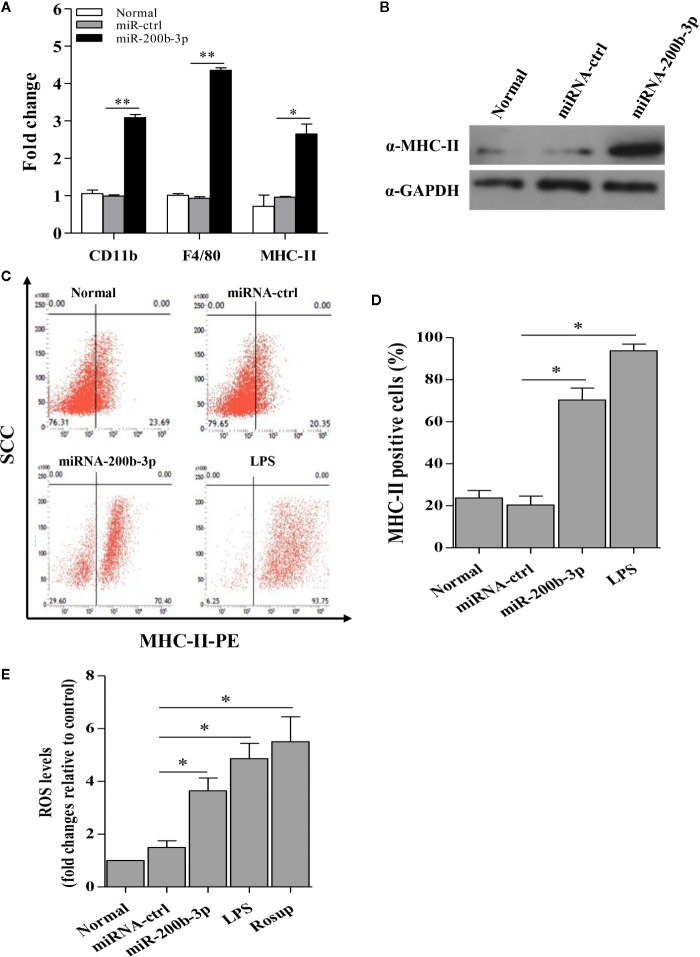
gga-miR-200b-3p promotes the activation and differentiation of HD11 cells. **(A)** Effect of gga-miR-200b-3p on the transcriptional levels of CD11b, F4/80, and MHC-II. **(B)** Effect of gga-miR-200b-3p on the expression of MHC-II. **(C, D)** HD11 cells were transfected with miRNA mimics, inhibitor or controls, and harvested for MHC-II staining for macrophage activation analysis using flow cytometry. **(E)** Effect of gga-miR-200b-3p on the production of reactive oxygen species (ROS) in HD11 cells. Data are representative of three independent experiments and presented as mean ± SD. miR-ctrl represents the negative control of gga-miR-200b-3p. ** stands for *P* < 0.01 and * for *P* < 0.05.

Since miR-200b-3p plays a critical role in the regulation of macrophage differentiation in mammals (19), it was essential to determine whether miR-200b-3p involves macrophage activation and differentiation in birds. Considering CD11b and F4/80 are the macrophage surface markers, we examined the effect of gga-miR-200b-3p on the expression of CD11b and F4/80 in HD11 cells. As a result, compared to that of the control group, the transcription of CD11b and F4/80 were significantly increased in miRNA mimic-treated cells. In contrast, miRNA inhibitor effectively lowered the transcriptional levels of CD11b and F4/80 (**Figure 2A**), indicating that gga-miR-200b-3p promotes macrophage activation in birds.

Because the expression of MHC-II in the cytoplasm is a typical characteristic during the macrophage activation, we proposed that gga-miR-200b-3p might enhance the expression of MHC-II. Therefore, we examined the effect of gga-miR-200b-3p on MHC-II expression using diverse assays. As a result, compared to that of the control group, over-expression of gga-miR-200b-3p mimics in HD11 cells effectively enhanced the transcriptional levels and expression level of MHC-II (**Figures 2A, B**). To consolidate this finding, we examined the effect of gga-miR-200b-3p on MHC-II expression using Flow cytometry assay. Interestingly, we found that over-expression of gga-miR-200b-3p mimics markedly enhanced the expression level of MHC-II in HD11 cells (**Figures 2C, D**). Considering the production of ROS during macrophage activation, we wondered whether gga-miR-200b-3p affects the intracellular ROS level in HD11 cells. As a result, gga-miR-200b-3p effectively enhanced the production of ROS in HD11 cells (**Figure 2E**). All these data provided solid evidence for the positive effect of gga-miR-200b-3p on macrophage activation in birds.

**Figure 3:**
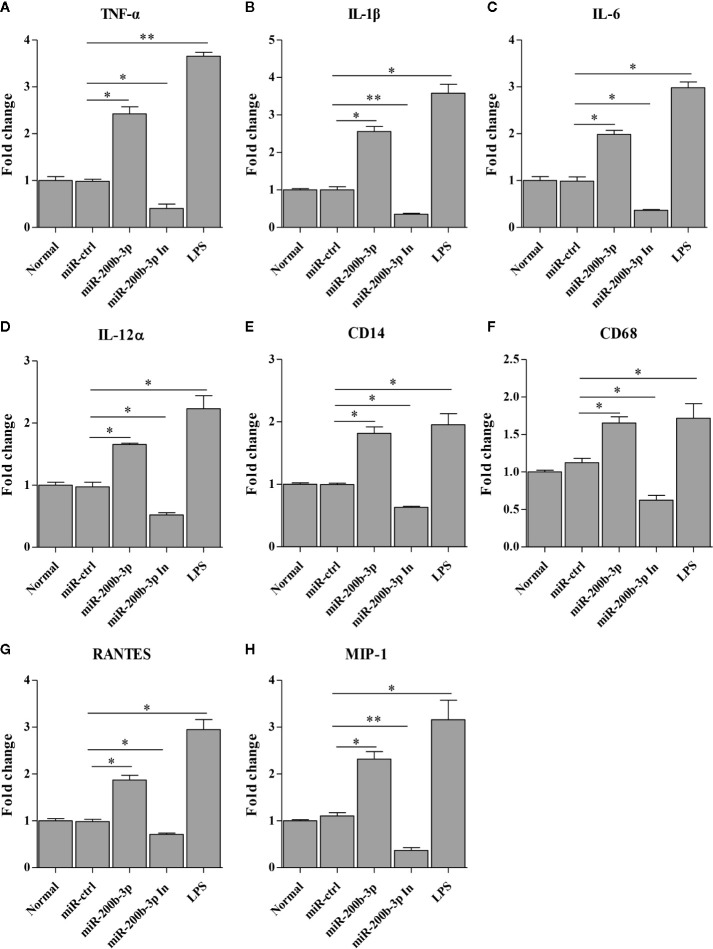
gga-miR-200b-3p promotes the transcription of cytokines in HD11 cells. HD11 cells were transfected with miRNA mimics, inhibitor, or controls. Twenty-four hours after transfection or treatment, cell cultures were harvested, and the transcriptional levels of TNF-α **(A)**, IL-1β **(B)**, IL-6 **(C)**, IL-12α **(D)**, CD14 **(E)**, CD68 **(F)**, RANTES **(G)**, and MIP-1 **(H)** were determined by qRT-PCR assay. Data are representative of three independent experiments and presented as mean ± SD. The miR-ctrl and miR-200b-3p In represent the negative control and the inhibitor of miR-200b-3p, respectively. ** stands for *P* < 0.01 and * for *P* < 0.05.

**Figure 4:**
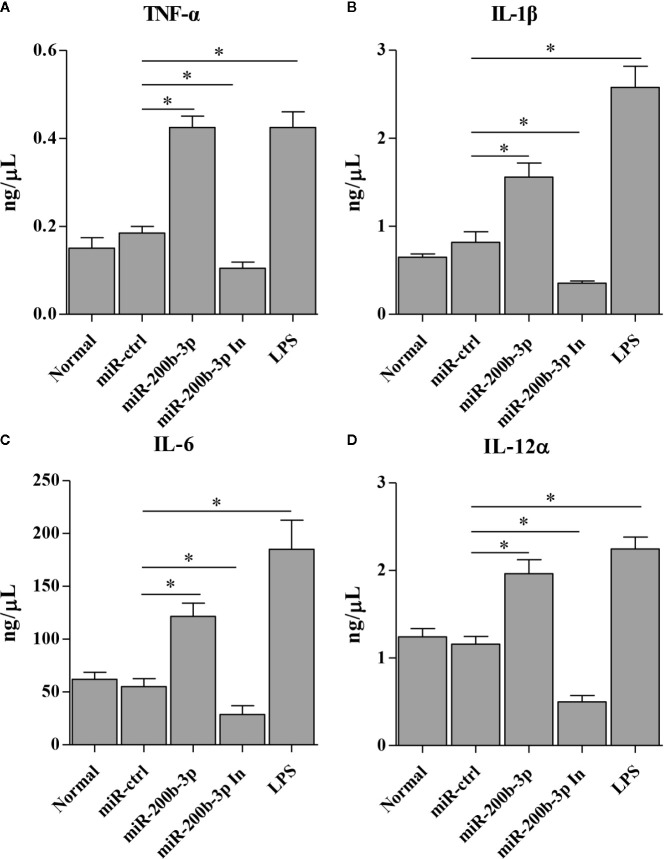
gga-miR-200b-3p promotes the expression of pro-inflammatory cytokines in HD11 cells. HD11 cells were transfected with miRNA mimics, inhibitor, or controls. Twenty-four hours after transfection or treatment, cell supernatants were harvested and the expression of TNF-α **(A)**, IL-1β **(B)**, IL-6 **(C)**, and IL-12α **(D)** were determined by ELISA. Data are representative of three independent experiments and presented as mean ± SD. miR-ctrl and miR-200b-3p In represent the negative control and the inhibitor of miR-200b-3p, respectively. * stands for *P* < 0.05.

The gga-miR-200b-3p enhances macrophage activation in birds, and the activated macrophage can secret pro-inflammatory cytokines and chemokines. This led us to examine the effect of gga-miR-200b-3p on the expression of pro-inflammatory cytokines and chemokines in HD11 cells. Thus, we detected the production of cytokines in HD11 cells with or without gga-miR-200b-3p treatment using qRT-PCR and ELISA. As a result, the transcriptional levels of TNF-α, IL-1β, IL-6, IL-12α, CD14, CD68, RANTES, and MIP-1 were significantly increased in miRNA mimic-treated HD11 cells, but decreased in miRNA inhibitor-treated HD11 cells (**Figures 3A–H**). To further substantiate the positive role of gga-miR-200b-3p in promoting the expression of pro-inflammatory cytokines, ELISA was employed in this study. As a result, the expression of TNF-α, IL-1β, IL-6, and IL-12α were significantly increased in HD11 cells transfected with gga-miR-200b-3p (**Figures 4A–D**), yielding the trend similar to that determined by qRT-PCR. All these data strongly suggested that gga-miR-200b-3p enhances macrophage activation and differentiation in birds.

### gga-miR-200b-3p Directly Targets MMD

**Figure 5:**
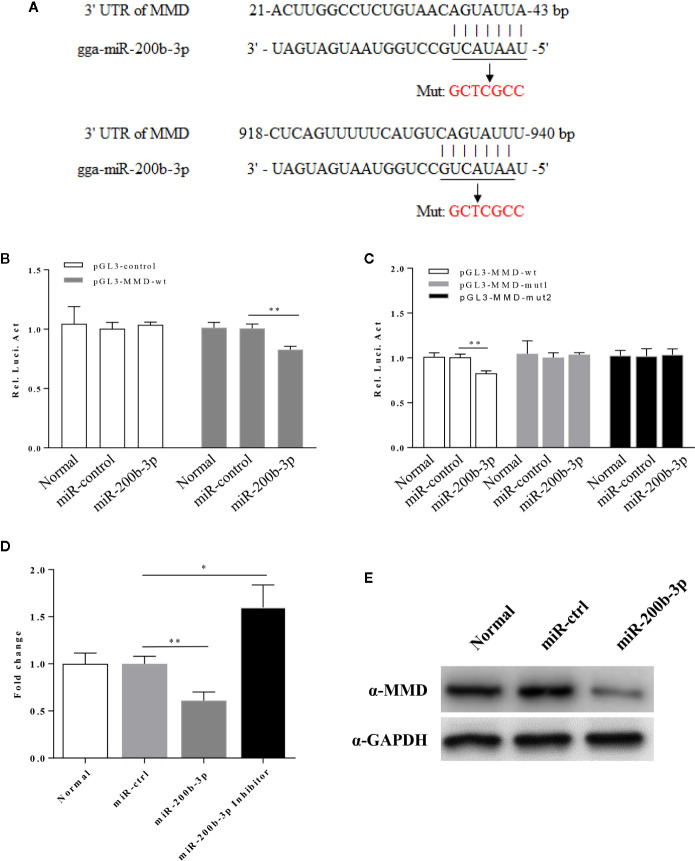
gga-miR-200b-3p directly targets MMD. **(A)** Diagram of predicted target sites for gga-miR-200b-3p in the MMD gene. The seed sequences of gga-miR-200b-3p are underlined and were mutated as indicated by the arrow. **(B)** Transfection of gga-miR-200b-3p reduced expression of MMD. The relative level of luciferase activity was calculated as follows: luciferase activity of reporter plasmid-transfected cells or cells co-transfected with the reporter plasmid and miRNA mimics/luciferase activity of cells co-transfected with the WT reporter plasmid and miRNA controls. **(C)** The mutation of the target site abolished the inhibition of MMD by gga-miR-200b-3p. **(D, E)** gga-miR-200b-3p inhibits the expression of MMD. Data are representative of three independent experiments and presented as mean ± SD. ** stands for *P* < 0.01 and * for *P* < 0.05.

Using the prediction software mentioned above, we found a putative gga-miR-200b-3p targeted gene MMD that likely modulate macrophage activation and differentiation. Since miRNA usually serves as a guide RNA to target mRNAs to degrade mRNAs or inhibit translation, it was logical to examine the relation of gga-miR-200b-3p with MMD. Therefore, we constructed firefly luciferase reporter pGL3-MMD-wt (21-43 and 918-940), and two mutant plasmids pGL3-MMD-mut1 (21-43) and pGL3-MMD-mut2 (918-940) with mutations in the seed regions (**Figure 5A**), and then transfected DF-1 cells with these reporter plasmids and miRNA mimics. As a result, gga-miR-200b-3p markedly inhibited the luciferase activities of pGL3-MMD-wt (**Figure 5B**), but do not affect the luciferase activities of pGL3-MMD-mut (**Figure 5C**), indicating that MMD is targeted by gga-miR-200b-3p. Moreover, overexpression of epigenetic gga-miR-200b-3p reduced the expressions of MMD at both mRNA and protein levels (**Figures 5D, E**). All these data indicated that MMD is targeted and regulated by gga-miR-200b-3p.

### MMD Expression Is Down-Regulated During Macrophage Activation

**Figure 6:**
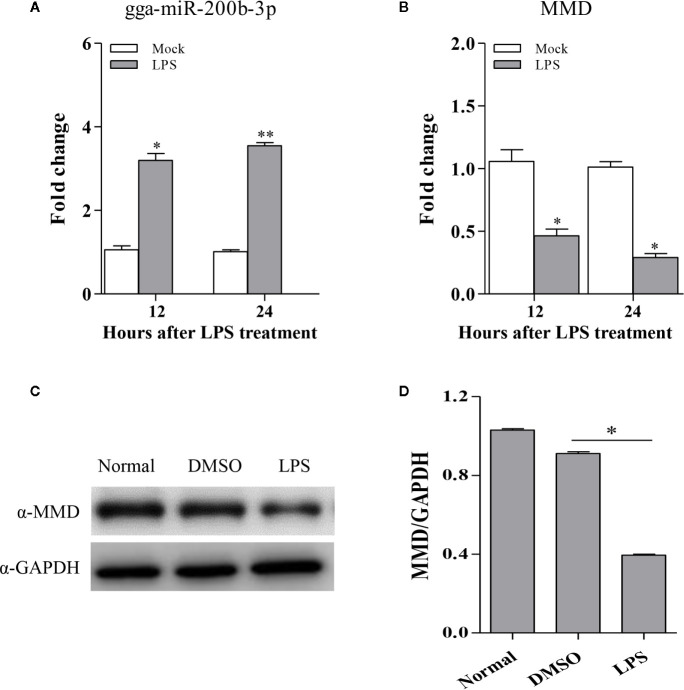
The expression of gga-miR-200b-3p and MMD in HD11 cells with LPS stimulation. HD11 cells were treated with LPS and harvested after 24 or 48 h treatment. **(A, B)** Total RNA was isolated from the cells for examining the expression of miR-200b-3p and MMD. **(C, D)** the expression of MMD was analyzed using Western Blot. Results are representative of three independent experiments and presented as mean ± SD. ** stands for *P* < 0.01 and * for *P* < 0.05.

The fact that vaccination-induced upregulation of gga-miR-200b-3p in birds, gga-miR-200b-3p directly targets MMD, and miR-200b-3p plays a critical role in the regulation of macrophage activation in mammals (19) prompted us to investigate the possibility that the expression of gga-miR-200b-3p and MMD are related to macrophage activation in birds. Considering the function of LPS on triggering the macrophage activation and differentiation, we examined the expression of gga-miR-200b-3p and MMD in HD11 cells treated with LPS. As a result, gga-miR-200b-3p was markedly upregulated in HD11 cells upon LPS stimulation (**Figure 6A**). Interestingly, consistent with a previous report (27), the expression of MMD was down-regulated at mRNA and protein levels after LPS stimulation (**Figures 6B–D**). All these results suggest that macrophage activation is highly correlated to the expression of gga-miR-200b-3p and MMD in birds.

### Knockdown of MMD Enhanced Macrophage Activation and Differentiation

**Figure 7:**
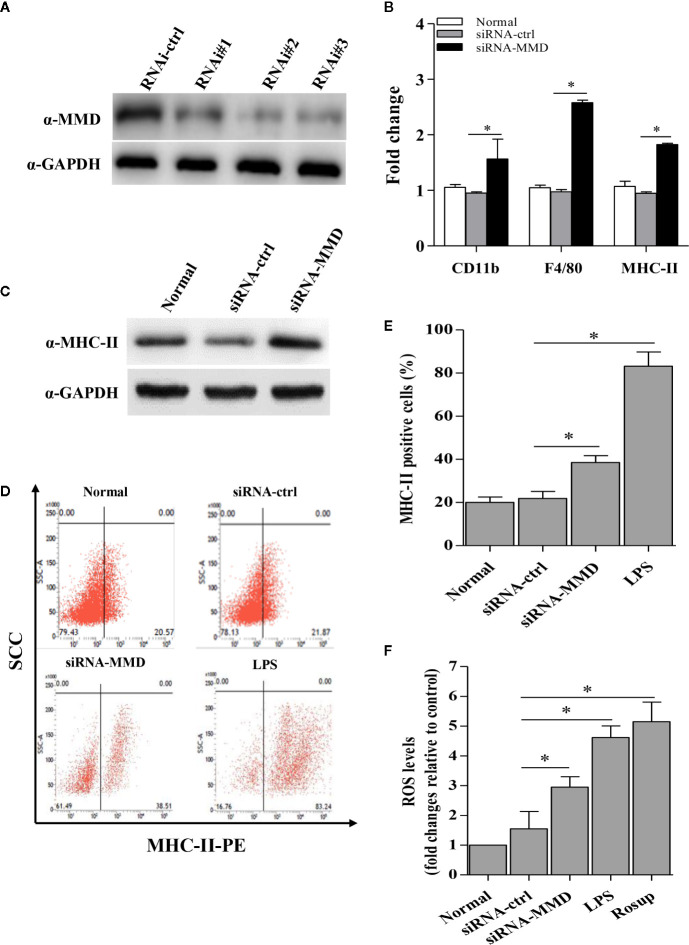
The inhibition of MMD by gga-miR-200b-3p enhanced macrophage activation and differentiation. **(A)** Effects of MMD RNAi on the expression of endogenous MMD in HD11 cells. **(B)** The knockdown of MMD enhances the transcriptional levels of CD11b, F4/80, and MHC-II. **(C, D)** HD11 cells receiving RNAi constructs were harvested for MHC-II staining for macrophage activation analysis using flow cytometry. **(E)** The knockdown of MMD promotes the expression of MHC-II. **(F)** The knockdown of MMD affects the production of reactive oxygen species (ROS). Data are representative of three independent experiments and presented as mean ± SD. The siRNA-ctrl represents the negative control of siRNA-MMD. * stands for P < 0.05.

**Figure 8:**
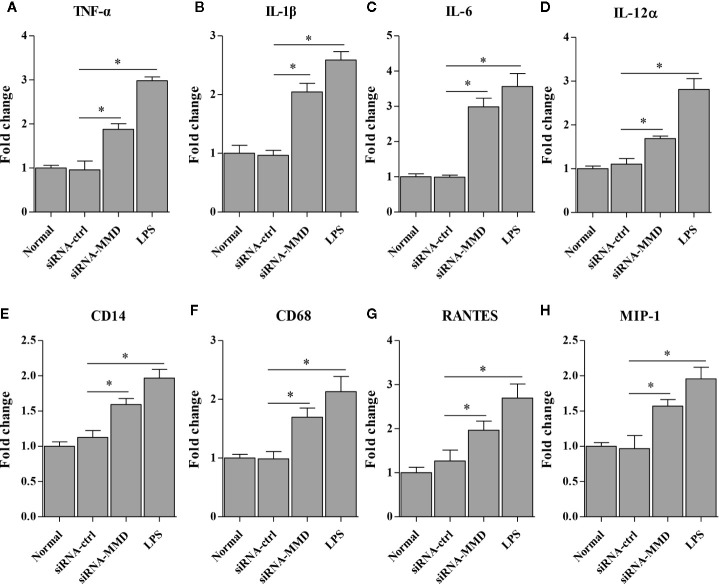
The knockdown of MMD promotes the transcriptional levels of cytokines in HD11 cells. HD11 cells transfected with RNAi or controls were harvested for the measurement of TNF-α **(A)**, IL-1β **(B)**, IL-6 **(C)**, IL-12α **(D)**, CD14 **(E)**, CD68 **(F)**, RANTES **(G)** and MIP-1 **(H)** using the qRT-PCR assay. Data are representative of three independent experiments and presented as mean ± SD. siRNA-ctrl represents the negative control of siRNA-MMD. * stands for *P* < 0.05.

**Figure 9:**
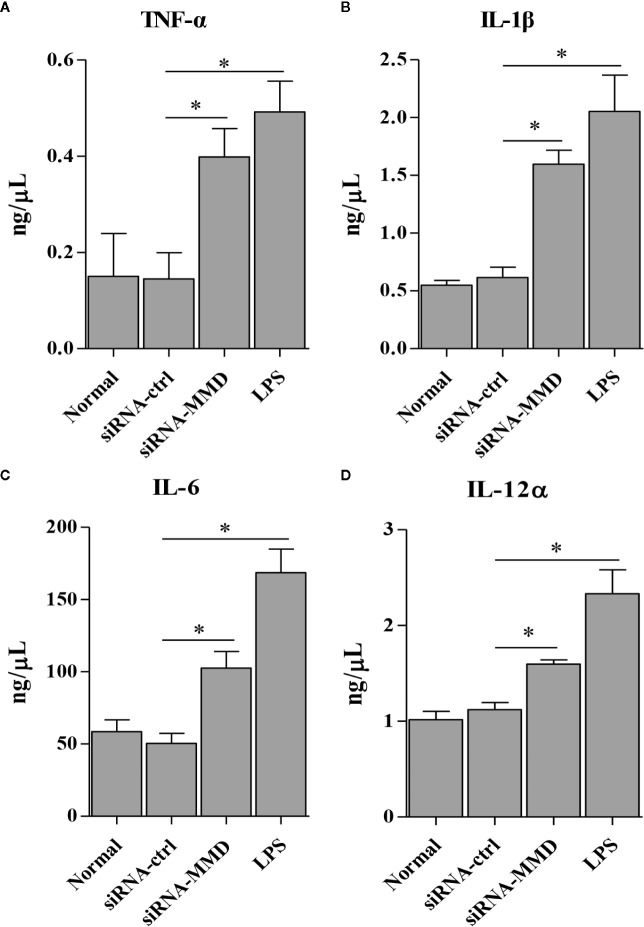
The knockdown of MMD promotes the expression of cytokines in HD11 cells. HD11 cells transfected with RNAi or controls, were harvested for the measurement of TNF-α **(A)**, IL-1β **(B)**, IL-6 **(C)**, and IL-12α **(D)** using ELISA assay. Data are representative of three independent experiments and presented as mean ± SD. siRNA-ctrl represents the negative control of siRNA-MMD. * stands for P < 0.05.

To examine the role of MMD in the macrophage activation and differentiation in birds, we made three MMD RNAi constructs. We identified one construct that could effectivelyreduce the cellular level of MMD without causing any cytopathic effects (**Figure 7A**). We subsequently examined the expression of marker molecules in HD11 cells receiving siRNA or control siRNA. As a result, knockdown of MMD effectively enhanced the transcriptional levels of CD11b and F4/80 in HD11 cells (**Figure 7B**); the low level expression of MMD markedly enhanced MHC-II expression at both mRNA and protein levels in HD11 cells (**Figures 7B, C**). We also examined the effect of MMD on MHC-II expression in HD11 cells using Flow cytometry assay. As a result, knockdown of MMD by RNAi effectively enhances the expression of MHC-II and activates the macrophage (**Figures 7D, E**). The intracellular ROS levels were detected in HD11 cells receiving this siRNA or control siRNA. As expected, cells with lower expression of MMD produced more ROS (**Figure 7F**). These data provide clues that MMD plays a critical role during macrophage activation in birds. To further substantiate the regulation of MMD on macrophage activation and differentiation, we examined the effect of MMD on the expression of pro-inflammatory cytokines, surface markers, and chemokines in HD11 cells receiving siRNA or control siRNA. As expected, knockdown of MMD by RNAi effectively increased the expression of TNF-α, IL-1β, IL-6, IL-12α, CD14, CD68, RANTES, and MIP-1 at mRNA level in HD11 cells (**Figure 8**). Furthermore, knockdown of MMD by RNAi significantly enhanced the expression of TNF-α, IL-1β, and IL-6, IL-12α at protein level (**Figure 9**). As gga-miR-200b-3p inhibited MMD expression, these data strongly suggested that gga-miR-200b-3p enhances macrophage activation and differentiation *via* degradation of MMD.

## Discussion

Macrophages are well known for their pivotal roles both in innate and adaptive immunity (28, 29). Macrophages can be activated by various stimuli, such as viruses, microbes, parasites, lipoproteins (LPS), cytokines, and apoptotic or necrotic cells (30–33). Then the activated macrophages play roles in pathogen clearance, immunomodulatory effects, and tissue integrity maintenance (34). Macrophages are usually classified into two subgroups: M1 and M2 (28, 35). M1 macrophages triggered by TNF-α or LPS are proinflammatory, playing central roles in host defense against pathogen infection. The polarization of M1 macrophages enhances the microbicidal or tumoricidal capacity through production of ROS and nitric oxide (NO). It facilitates immune responses by producing proinflammatory cytokines such as IL-12 and TNF-α. In contrast, M2 macrophages are related to the responses to helminth infection, chronic inflammation, tissue remodeling, and cancer through enhancing the secretion of anti-inflammatory cytokines IL-10 (27, 30).

HD11 cells were developed by a transformation of the chicken bone marrow cells with a replication-defective retrovirus, possessing functions and surface markers of typical macrophage (20, 36). Till now, HD11 cells have been widely used for the research of infection and immunity in birds (20, 26, 36–38). Considering the macrophage characteristics of the cell lines, we employed HD11 cells as a cellular model to screen whether and which miRNAs involve the activation and differentiation of macrophage in birds.

It has been reported that several miRNAs are essential regulators during macrophages activation and differentiation in a mammal, such as miR-424, miR-223, miR-155, miR-142-3p, and miR-200b-3p (19, 39–42). MiR-424 can be activated by the master transcription factor PU.1 subsequently suppressing the translation of NFI-A; the interlinked components PU.1/miR-424/NFI-A play critical roles in regulating monocyte/macrophage differentiation (39). MiR-223 could negatively regulate the proliferation of progenitor, and the activation and differentiation of granulocyte by targeting Mef2c, a transcription factor that promotes myeloid progenitor proliferation (40). MiR-155 drives granulocyte/monocyte expansion when constitutively expressed in mouse hematopoietic stem cells, and also directly represses a series of target genes associated with myeloid hyperplasia and/or hematopoiesis (41). MiR-142-3p promotes macrophage differentiation and affects immune-suppressive function in cancer cells through down-regulating gp130 and repressing C/EBPβ LAP (42). MiR-200b-3p enhances the activity of p38 MAPK by targeting p38IP, hence promotes G1/S arrest and monocyte/macrophage differentiation (19). However, few reports regarding the effect of miRNAs on macrophage activation and differentiation in birds are available. This study aimed to screen the potential host miRNAs regulating macrophage activation and differentiation in chickens.

MiR-200b-3p plays a critical role in many biological processes, such as cell proliferation, apoptosis, and metastasis. MiR-200b-3p regulates cell proliferation and apoptosis by targeting of Sp1 transcription factor (SP1) (43), induces apoptosis by regulating NF-κB pathway in BC cells (44), regulates CRC progression and metastasis by modulating the expression of ZEB1 (45), and regulates the self-renewing divisions by lessening Notch signaling in pancreatic cancer stem cells (46). Interestingly, a recent report indicated that miR-200b-3p is a novel player in regulating monocyte/macrophage differentiation in humans (19). Thus, it is intriguing to analyze the effect of miR-200b-3p on macrophage in birds. Interestingly, in this study, we found the positive effect of gga-miR-200b-3p on macrophage activation and differentiation in chickens. These data indicated that miR-200b-3p might promote macrophage activation and differentiation regardless of animal species. More effort will be required to analyze the effect of miR-200b-3p on macrophage activation and differentiation in other animals.

Mounting evidence suggests that microRNAs play critical roles in regulating post-transcriptional translation through regulating their target genes (47). In his study, we reported that MMD is a critical downstream target of gga-miR-200b-3p and this conclusion is supported by the following evidence: (1) the conserved complementary sequence of gga-miR-200b-3p is identified in the 3′UTR of MMD mRNA; (2) overexpression of gga-miR-200b-3p markedly decreased the activity of a luciferase reporter containing the wild type 3′UTR of MMD, but did not affect the luciferase reporter containing the mutant 3′UTR of MMD; (3) overexpression of gga-miR-200b-3p significantly suppressed MMD expression, whereas inhibition of gga-miR-200b-3p enhanced MMD expression; (4) the effect of gga-miR-200b-3p on macrophage activation and differentiation was reversed by knockdown of MMD; (5) the trends of MMD and gga-miR-200b-3p in LPS-treated HD11 cells are similar to that determined by deep sequencing. Together, these data strongly suggest that gga-miR-200b-3p enhances macrophage activation and differentiation through downregulating MMD in birds. However, based on results from prediction using bioinformatic prediction, other genes may act as the target genes of gga-miR-200b-3p. Still, we did not have any solid evidence for the presence of other specific target genes. Of course, it is still possible that gga-miR-200b-3p targets more genes than expected. More effort will be required to focus on the target genes of gga-miR-200b-3p in the future.

MMD was first identified in humans in 1995 and preferentially expressed in mature macrophages (48). Later, the MMD orthologue (macrophage/microglia activation factor, MAF), was identified in lesion-associated microglial cells in rats (49, 50). MMD belongs to the progesterone and adipoQ receptor (PAQR) family, and contains a putative conserved 7 transmembrane motif (51) suggested that MMD might play a vital role in the cellular process *via* effecting on some primary signaling pathways (27). MMD is usually localized at the endoplasmic reticulum, mitochondria, and Golgi apparatus, playing an oncogenic role. The knockdown of MMD promoted cell cycle arrest and inhibited the growth of A549 and LLC lung cancer cell lines (27). Interestingly, MMD is implicated in macrophage activation (27, 48). The previous study showed that overexpression of MMD in macrophages increases the production of TNF-α and NO, and enhances ERK1/2 and Akt phosphorylation upon LPS stimulation (27), indicating that MMD might increase the production of TNF-α and NO in LPS-stimulated macrophages through ERK1/2 and Akt phosphorylation. However, the exact biological function of MMD is still unclear. More effort will be required to study the underlying mechanism of MMD regulating the macrophage activation in the future.

Multiple lines of evidence have shown that macrophage activation and differentiation are characterized by the expression of F4/80 and CD11b on the plasma membrane, cytokines secretion, expression of MHC in the cytoplasm, and production of NO (52, 53). The markers on the macrophage surface, F4/80 and CD11b, are usually used to judge the activation status of macrophages. However, we cannot analyze the expression of both molecules using Western Blot and Flow cytometry due to the limitation of reagents, especially the antibodies against chicken F4/80 and CD11b. Thus, we just analyzed the transcriptional levels of CD11b and F4/80 with epigenetic upregulation of gga-miR-200b-3p expression or knockdown the expression of MMD in HD11 cells. As professional antigen-presenting cells (APC), activated macrophages could process antigenic peptides, express MHC-II, and present to cognate T cells for priming and activation (54). So gga-miR-200b-3p was assumed to affect the expression of MHC-II in HD11 cells. To test this hypothesis, we examined the expression level of MHC-II in HD11 cells receiving miRNA mimics or control, siRNA or control using qRT-PCR, Western Blot, and Flow cytometry. Consistent with these characteristics of macrophage activation and differentiation, in this study, epigenetic upregulation of gga-miR-200b-3p expression or knockdown the expression of MMD in HD11 cells enhances the expression levels of MHC-II. Considering the production of pro-inflammatory cytokines during macrophage activation and differentiation, gga-miR-200b-3p might affect the expression of pro-inflammatory cytokines. Our findings also confirmed this hypothesis: epigenetic upregulation of gga-miR-200b-3p expression or knockdown the expression of MMD in HD11 cells enhanced the expression of pro-inflammatory cytokines (TNF-α, IL-1, IL-6 and IL-12), chemokines (RANTES and MIP-1) and cell surface markers (CD14 and CD68), and the production of ROS. All these data provided solid evidence that gga-miR-200b-3p enhances macrophage activation and differentiation *via* reducing the expression of MMD.

In summary, this is the first study to demonstrate the effect of miRNAs on macrophage activation and differentiation in birds. In this study, we found that gga-miR-200b-3p effectively enhances the macrophage activation and differentiation in birds, and MMD has an overall negative effect on macrophage activation and differentiation. Our findings strongly suggest that gga-miR-200b-3p markedly enhances macrophage activation and differentiation *via* decreasing the expression of MMD.

## Data Availability Statement

The raw data supporting the conclusions of this article will be made available by the authors, without undue reservation, to any qualified researcher.

## Ethics Statement

The animal study was reviewed and approved by the Animal Care Committee of South China Agricultural University (approval ID: SYXK-2014-0136).

## Author Contributions

QL, QX, XZ, and WL designed the study, interpreted the data, and gave final approval of the version to be published. WL, LZ, ML, DZ, and YY performed the experiments and data collection. Y-FC contributed to data analysis and manuscript revision. All authors contributed to the article and approved the submitted version.

## Funding

This work was supported by the Guangdong Provincial Promotion Project on Preservation and Utilization of Local Breed of Livestock and Poultry (4000-F18260), National Natural Science Foundation of China (31802206), the Natural Science Foundation of Guangdong Province (2018A030310206), and Guangdong Province Modern Agricultural Industry Technology System Project (2019KJ128). 

## Conflict of Interest

The authors declare that the research was conducted in the absence of any commercial or financial relationships that could be construed as a potential conflict of interest.
